# Periplanetasin-2 Enhances the Antibacterial Properties of Vancomycin or Chloramphenicol in *Escherichia coli*

**DOI:** 10.4014/jmb.2010.10058

**Published:** 2020-11-27

**Authors:** Heejeong Lee, Jae Sam Hwang, Dong Gun Lee

**Affiliations:** 1 School of Life Sciences, BK2 Four KNU Creative BioResearch Group, Kyungpook National University, Daegu 4566, Republic of Korea; 2 Department of Agricultural Biology, National Academy of Agricultural Science, RDA, Wanju 55365, Republic of Korea

**Keywords:** Periplanetasin-2, vancomycin, chloramphenicol, synergistic interaction

## Abstract

Periplanetasin-2 from cockroach exhibits broad-spectrum antimicrobial activity. The underlying antibacterial mechanisms rely on the stimulation of reactive oxygen species overproduction to induce apoptotic cell death. A promising strategy to increase the bioavailability of periplanetasin-2 involves reducing the dose through combination therapy with other antibacterials that show synergistic effects. Thus, the synergistic antibacterial activity of periplanetasin-2 with conventional antibacterial agents and its mechanisms was examined against *Escherichia coli* in this study. Among the agents tested, the combinations of periplanetasin-2 with vancomycin and chloramphenicol exhibited synergistic effects. Periplanetasin-2 in combination with vancomycin and chloramphenicol demonstrated antibacterial activity through the intracellular oxidative stress response. The combination with vancomycin resulted in the enhancement of bacterial apoptosislike death, whereas the combination with chloramphenicol enhanced oxidative stress damage. These synergistic interactions of periplanetasin-2 can help broaden the spectrum of conventional antibiotics. The combination of antimicrobial peptides and conventional antibiotics is proposed as a novel perspective on treatments to combat severe bacterial infection.

## Introduction

Increasing rates of life-threatening infections due to not only multidrug-resistant gram-negative bacteria but also pathogens that are resistant to all current therapeutic options have been reported [1]. To overcome this progression of bacterial resistance and treat drug-resistant infection, strategies to develop new and effective antibiotics are urgently needed [2]. The number of candidates currently in development is insufficient to control this global threat and the pace of research is slow due to many challenges [1, 3]. With no reprieve from therapeutic reliance on the current antibiotic pipeline, bridging the gap between widespread bacterial resistance and the development of new antibiotics will require the creative use of available treatment options [4]. One approach to control bacterial infection is combination therapy in which antibiotics are administered together with other antimicrobial or non-antimicrobial agents. The purpose of using combination antimicrobial therapy is to prevent or delay the emergence of resistance during treatment [5, 6]. For instance, synergistic interactions between anti-inflammatory agents and antibiotics can potentially prevent the emergence of resistance, increase antibacterial efficacy, and provide broader-spectrum antibacterial activity than antibiotic monotherapy [3].

With epidemic levels of antibiotic resistance established or emerging across an increasingly broad spectrum of human pathogens, antimicrobial peptides (AMPs) have become especially attractive due to their unique mechanism of action, namely the disruption of bacterial membrane integrity [4]. Natural AMP-based defense systems have evolved to act synergistically against microorganisms in the host environment. Synergism between AMPs and antibiotics has evolved as a natural strategy to ensure host protection against a broad spectrum of pathogens, thereby explaining the presence of a wide array of AMPs within a single host [7]. Periplanetasin-2 exerts its antifungal activity via apoptotic cell death and antibacterial activity including membrane disruption and bacterial apoptotic death [8, 9]. Herein, the effects of periplanetasin-2 in combination with various antibiotics on *Escherichia coli* were investigated.

## Materials and Methods

### Compounds, Bacterial Strains, and Culture Conditions

Periplanetasin-2 was purchased from Anygen Co. (Republic of Korea). The detailed procedure of peptide synthesis was described in a previous study [8]. Norfloxacin, chloramphenicol, vancomycin, amikacin, kanamycin, rifampicin, ciprofloxacin, cefotaxime, and hygromycin B were purchased from Sigma-Aldrich (USA). *Escherichia coli* wild type (WT) BW25113 was obtained from the Coli Genetic Stock Center. For all assays, bacterial cells were grown in Luria-Bertani (LB) broth (BD Biosciences, USA) under aerobic conditions at 37°C while shaking at 120 ×*g*. The cells were centrifuged and suspended in phosphate-buffered saline (PBS). Afterward, the cells were incubated with periplanetasin-2 or the conventional antibiotics at 37°C.

### Bacterial Susceptibility Test

A minimum inhibitory concentration (MIC) test was performed using the twofold standard broth-microdilution method following the Clinical and Laboratory Standards Institute (CLSI) guidelines [10]. The tested bacteria were diluted to 1 × 10^6^ CFU/ml. Diluted bacterial cells at the exponential phase were dispensed into microtiter plates (100 μl/well) and the test compounds were added. The MIC was determined after overnight incubation at 37°C by measuring the optical density at 600 nm using an ELx800 absorbance microplate reader (BioTek, USA).

### Checkerboard Assay

The interactions of periplanetasin-2 with conventional drugs were evaluated using the checkerboard method [11]. A two‐dimensional (2D) checkerboard with twofold serial dilutions of periplanetasin-2 and the conventional antibacterial agents was used for the study. Control wells containing medium were included in each plate. Growing cells were dispensed into 96-well plates (0.1 mL/well). Following incubation for overnight, cell growth was measured by monitoring the absorption at 600 nm using a microtiter ELISA Reader (BioTek Instruments). The interactions of periplanetasin-2 with the antibacterial agents were evaluated based on the fractional inhibitory concentration index (FICI), which was calculated using the following equation: FICI=FIC_A_+FIC_B_=(MIC_A comb_/MIC_A alone_)+(MIC_B comb_/MIC_B alone_), where MIC_A comb_ and MIC_B comb_ are the concentrations of drugs A and B that showed activity when combined and MIC_A alone_ and MIC_B alone_ are the concentrations of drugs A and B when acting alone, respectively. FICI, calculated as the sum of each FIC, was interpreted as follows: FICI ≤0.5 synergy, 0.5 < FICI ≤4 no interaction, 4 < FICI antagonism. Each test was performed in triplicate.

### Caspase Activation

Activation of RecA protein was detected using the CaspACE fluorescein isothiocyanate (FITC-VAD-FMK) in situ marker (Promega, USA). FITC-VAD-FMK, an FITC-conjugated peptide pan-caspase inhibitor, is transported into cells and binds to the active site of caspase as a substrate, indicating RecA expression. RecA has a classical binding site with the caspase substrate. The cells were incubated with periplanetasin-2 for 2 h at 37°C. Next, the cells were washed twice and incubated with CaspACE FITC-VAD-FMK for 30 min. After centrifugation, the cells were resuspended in PBS and the fluorescence was analyzed using a FACSVerse flow cytometer.

### Flow-Cytometric Analysis of Reactive Oxygen Species (ROS) Generation

*E. coli* cells were incubated with periplanetasin-2 (MIC or MIC for the combination of each antibiotic) and antibiotics (MIC or MIC for the combination of periplanetasin-2) for 2 h at 37°C. Flow cytometric estimation of ROS production was carried out in cells. Cells were stained with H_2_DCFDA in order to detect ROS respectively, as previously detailed. Cells were resuspended in 5 μM H_2_DCFDA. After incubation, cells were washed with PBS and relative fluorescence intensity was analyzed by FACSVerse flow cytometer (BD Biosciences).

### Lipid Peroxidation

Lipid peroxidation was quantified based on malondialdehyde (MDA) levels. After treatment with periplanetasin-2 for 2 h, the cell suspension was centrifuged at 12,000 ×*g* for 5 min. Then, the pellet was sonicated twice on ice in lysis buffer (2% Triton-X 100, 1% SDS, 100 mM NaCl, 10 mM Tris-HCl, and 1 mM EDTA pH 8.0). The mixture was centrifuged, and the supernatant was added to an equal volume of 0.5% (w/v) thiobarbituric acid (TBA) solution in 5% TCA. The mixture was heated at 95°C for 30 min and then cooled on ice. The absorbance of the reaction mixture was measured at 532 and 600 nm [12], and each experiment was performed in triplicate.

### Intracellular Calcium Ion Level Analysis

Intracellular calcium levels were assessed by cell‐permeant intracellular calcium indicator Fura 2 AM (Molecular Probes). When Fura 2 AM enters the cell, its acetoxymethyl groups are removed by cellular esterases and the calcium‐sensitive indicator Fura‐2 is generated. *E. coli* cells were incubated with periplanetasin-2 (MIC or MIC for the combination of each antibiotic) and antibiotics (MIC or MIC for the combination of AuNPs) for 2 h at 37°C (132 mM NaCl, 4 mM KCl, 1.4 mM MgCl_2_, 6 mM glucose, 10 mM HEPES, 10 mM NaHCO_3_, and 1 mM CaCl_2_, pH 7.4) and treated with 0.01% Pluronic F‐127 (Molecular Probes) and 1% bovine serum albumin. The samples were then incubated with 5 μM Fura‐2 AM at 37°C for 40 min. The cells were then washed with calcium‐free Krebs buffer (132 mM NaCl, 4 mM KCl, 1.4 mM MgCl_2_, 6 mM glucose, 10 mM HEPES, and 10 mM NaHCO_3_, pH 7.4) three times and analyzed using a spectrofluorophotometer (RF‐5301PC; Shimadzu, Japan) at wavelengths of 340 nm (excitation) and 510 nm (emission) [13].

### Measurement of Morphological Changes

To investigate morphological alteration by compounds, the changes in forward scatter (FSC) and side scatter (SSC) of bacterial cells were analyzed by flow cytometry. *E. coli* were incubated with periplanetasin-2 (MIC or MIC for the combination of each antibiotic), vancomycin and chloramphenicol (MIC or MIC for the combination of perplanetasin-2) for 2 h at 37°C. After incubation, the cells were washed with PBS. Bacterial cells were measured in each sample by determining their position on the FSC and SSC contour plots using a FACSVerse flow cytometer.

### Statistical Analysis

All experiments were performed in triplicate and the data were represented as the means ± SD. Statistical significance was determined via Student’s *t*-test. *p* < 0.05 was considered to indicate statistical significance.

## Results

### Antibacterial Activity of Periplanetasin-2 in Combination with Antibiotics

Periplanetasin-2 and several antibiotics exhibited antibacterial activity in *E. coli*, while vancomycin showed rare antibacterial activity against *E. coli* (Table 1). The antibacterial properties of periplanetasin-2 and conventional antibiotics had been investigated in a previous study. [9]. Here, we evaluated the effects of combinations of these antibiotics with periplanetasin-2 to inhibit bacterial growth. Synergistic effects were observed when periplanetasin-2 was applied in combination with chloramphenicol and vancomycin. No interactions were observed when periplanetasin-2 was combined with norfloxacin, ciprofloxacin, kanamycin, rifampicin, and cefotaxime. The combination with amikacin appeared to have an antagonistic effect. For periplanetasin-2 in combination with vancomycin or chloramphenicol, there were no antagonistic effects, but there was synergistic action (Table 2). The results showed that periplanetasin-2 has significant antibacterial activity when combined with vancomycin or chloramphenicol, expanding the antibacterial spectrum of vancomycin.

**Fig. 1:**
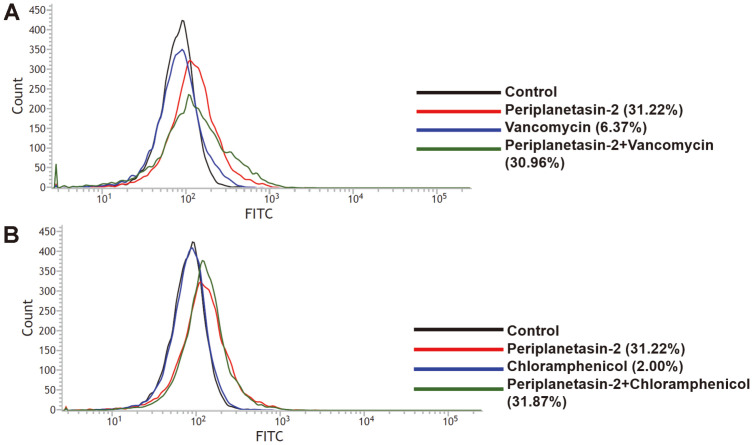
Effects of different drugs on apoptotic response in *E. coli*. Quantitation of caspase-like protein activation by FITC‐VAD‐FMK (**A**) Combination with periplanetasin-2 and vancomycin (**B**) Combination with periplanetasin-2 and chloramphenicol.

**The bacterial apoptotic features of periplanetasin-2 were enhanced by chloramphenicol and vancomycin.** Oxidative stress is associated with synergistic effects from metal nanoparticles and antibiotics [14]. Therefore, the relation with oxidative damage and a synergistic effect was observed. To investigate whether apoptosis-like death is induced by periplanetasin-2 during co-treatment with chloramphenicol and vancomycin, the expression of RecA protein was visualized using the FITC-conjugated pan caspase inhibitor, FITC-VAD-FMK [15]. The expression of RecA protein was observed by combining periplanetasin-2 with chloramphenicol or vancomycin. As shown in Fig. 1, the cells treated with periplanetasin-2 and a combination of vancomycin or chloramphenicol showed a significant increase in RecA expression compared to those treated with antibiotics alone. The increased percentage of cells stained with FITC-VAD-FMK was similar in the presence of periplanetasin-2 alone. These results suggest that apoptosis-like death induced by periplanetasin-2 is enhanced by vancomycin and chloramphenicol treatment.

**Fig. 2:**
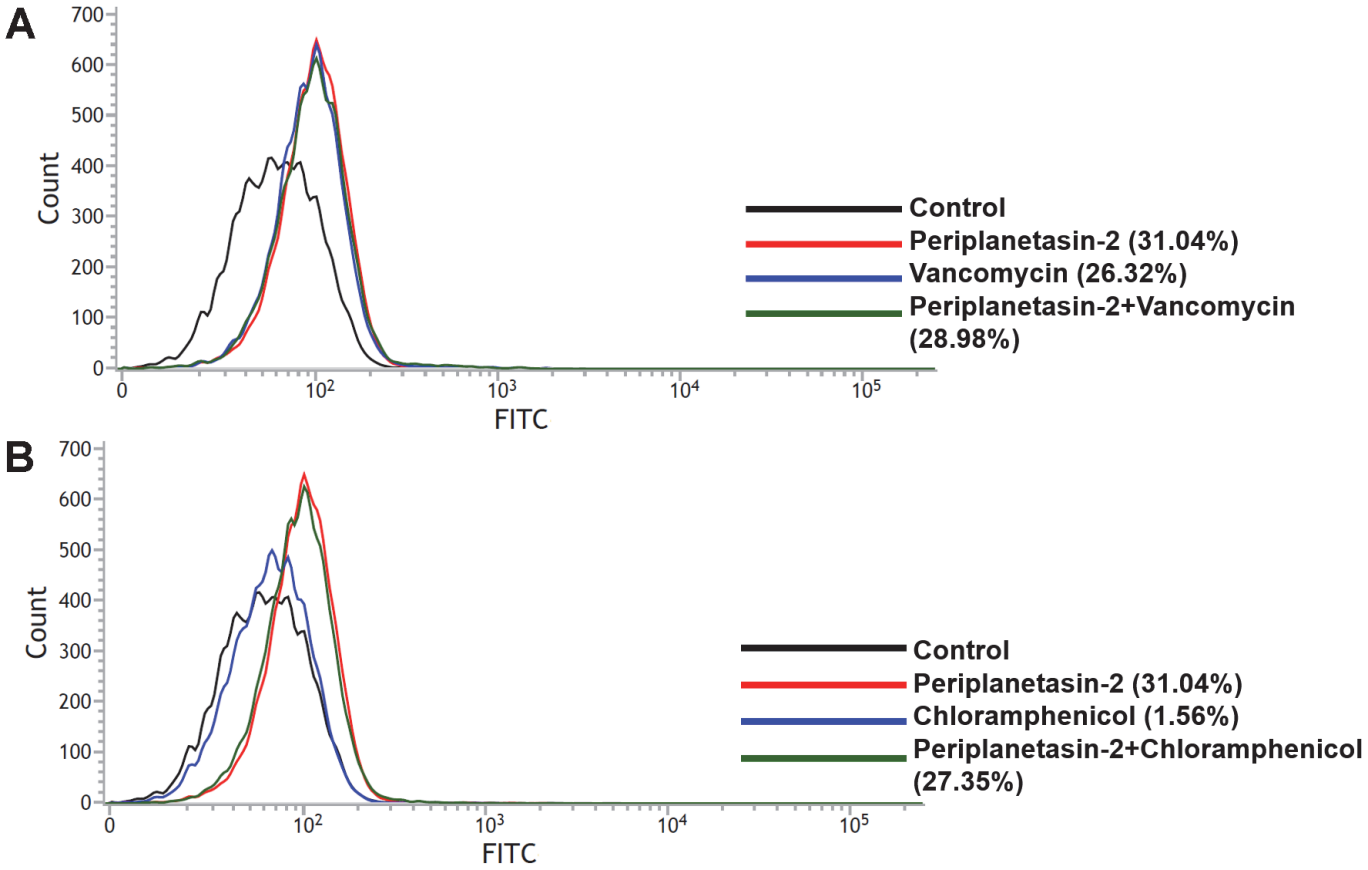
Effects of periplanetasin-2 on intracellular ROS accumulation in *E. coli*. Flow cytometric analysis of ROS accumulation was conducted using H_2_DCFDA.

Overproduction of ROS is the main cause of apoptosis-like bacterial cell death. To examine the synergistic effects of periplanetasin-2 combined with vancomycin or chloramphenicol, the involvement of ROS was investigated after application of the peptides individually and in combination. The increased fluorescence of H_2_DCFDA indicated the accumulation of ROS. Periplanetasin-2 alone caused ROS generation (31.04%) compared to the levels in untreated samples (13.39%), but the combination with vancomycin (28.98%) and chloramphenicol (27.35%) resulted in elevating ROS accumulation (Fig. 2). This result indicates that periplanetasin-2 exerts antimicrobial activity through mechanisms other than ROS production and exhibits a more potent effect with ROS induced by other antibiotics.

Changes in the oxidation state due to the accumulation of ROS can cause lipid peroxidation [16]. Therefore, cells were treated with periplanetasin-2, chloramphenicol, and vancomycin individually or in combination to investigate lipid peroxidation and the role of ROS in inducing apoptosis. Analysis of lipid peroxidation showed that the elevation of ROS caused an increase in the proportion of damaged bacterial cells compared with the damage caused by individual drugs. The exposure of cells to periplanetasin-2 increased lipid peroxidation compared to untreated cells. The combination with vancomycin or chloramphenicol increased the MDA levels. These results suggested that the compounds may have more potent effects due to the increased accumulation of ROS.

**Fig. 4:**
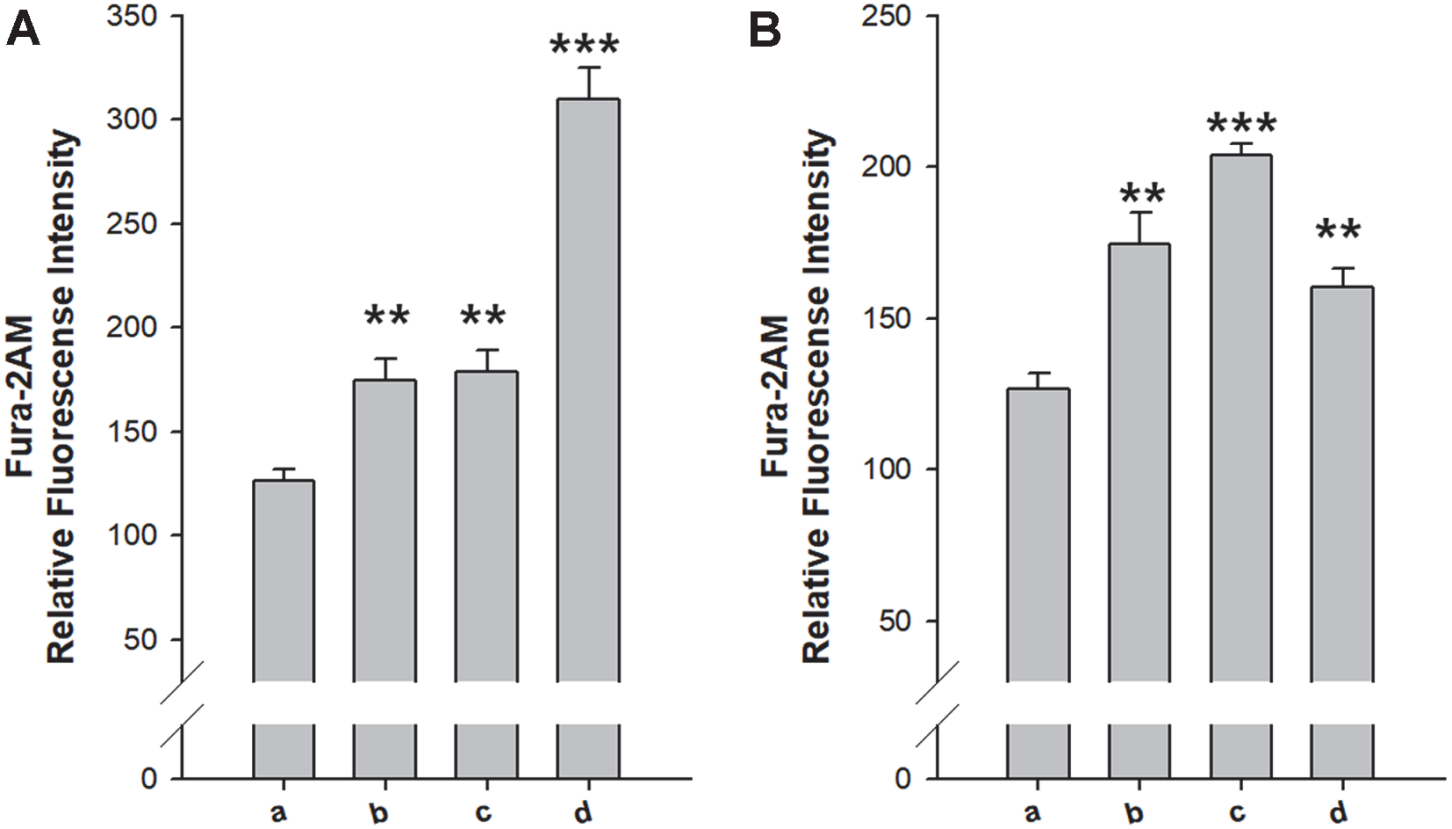
Periplanetasin-2 alters intracellular divalent cation concentration. *E. coli* cells were treated with periplanetasin-2, vancomycin, and chloramphenicol, alone or combined. (**A**) (a) Untreated (b) Periplanetasin-2 (c) Vancomycin (d) Periplanetasin-2+vancomycin (**B**) (a) Untreated (b) Periplanetasin-2 (c) Chloramphenicol (d) Periplanetasin- 2+chloramphenicol. Intracellular Ca^2+^ level was analyzed using Fura‐2 AM. Increased fluorescence of Fura‐2 AM indicates accumulation of Ca^2+^. The data represent the mean, standard deviation, and p values from three independent experiments (*p < 0.05; **p < 0.01; ***p < 0.001 vs. untreated samples).

**The mechanisms of action for the synergistic effects of vancomycin and chloramphenicol combined with periplanetain-2 are different.** The effect of periplanetasin-2 on the intracellular concentration of calcium when applied alone and combined with vancomycin or chloramphenicol was investigated. Intracellular calcium level was measured using Fura 2 AM and as shown in Fig. 4, periplanetasin-2 alone and in combination with vancomycin caused the accumulation of calcium. The elevation of calcium ions can be affected by the consolidation of apoptosis-like cell death. Interestingly, samples treated with periplanetasin-2 and chloramphenicol showed similar calcium levels compared to untreated cells. Therefore, it is possible that co-treatment of periplanetasin-2 and vancomycin increases calcium concentrations to stimulate apoptosis-like death signaling (Fig. 4). The combination of periplanetasin-2 and chloramphenicol could not stimulate calcium signaling. In other words, this combination simply elevated oxidative stress rather than enhancing apoptosis-like death signaling.

**Fig. 5:**
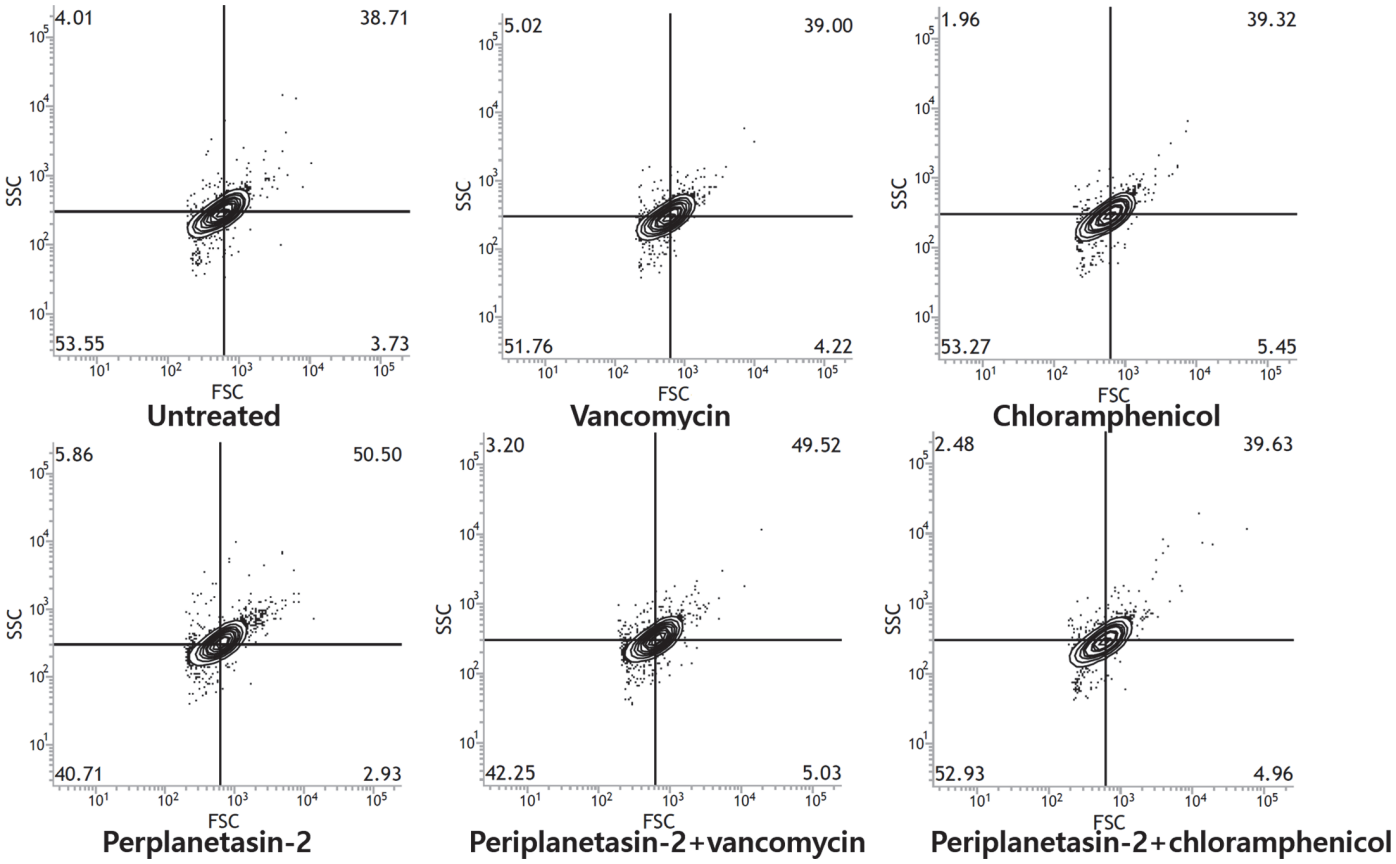
Periplanetasin-2 causes cell filamentation. *E. coli* cells were treated with periplanetasin-2, vancomycin and chloramphenicol, alone or combined. Morphological change was indicated by periplanetasin-2 and vancymycin co-treatment. Morphological change was not indicated by periplanetasin-2 and chloramphenicol co-treatment. Cell size was analyzed using flow cytometry. The indicated values refer to the percentage of cells in relation to the respective total number of cells. An increase in FSC and SSC indicates filamentation of the cells.

ROS react with different components of DNA to damage it by inducing lesions of sugars and bases, causing DNA strand breaks [17, 18]. Bacterial cells are observed to form filaments in response to DNA damage, antibiotic treatment, host immune systems, temperature, starvation, and other conditions related to clinical settings and food preservation [19]. Cell filamentation was observed when periplanetasin-2 was applied alone and in combination with vancomycin. The individual morphological change was characterized by apoptosis-like death (Fig. 5). These results indicated that periplanetasin-2 alone and in combination with vancomycin causes apoptosis-like death.

## Discussion

Severe bacterial infection may be minimized by a combination antibiotic regimen, in which the sensitivity of the results is evaluated following treatment. Additionally, combination therapy has been shown to yield improved results compared with a single treatment, and combination empirical antimicrobial therapy directed against gram-negative bacteria may be a more appropriate treatment approach than monotherapy [20, 21]. Within current antimicrobial and anti-resistance research, antimicrobial combinations involving antimicrobial peptides attract considerable attention [22]. Periplanetasin-2 exhibits potent antimicrobial activity against several microbial pathogens without hemolysis (data not shown). Particularly, the antibacterial mechanism of periplanetasin-2 is apoptosis-like death, which is mediated by excessive ROS generation. Several apoptotic hallmarks, such as membrane depolarization, DNA fragmentation, caspase-like protein activation, and phosphatidylserine exposure, are induced by periplanetasin-2 [8, 9]. In this study, we examined whether periplanetasin-2 has potential as a supplemental agent to improve clinical antibiotics.

The fluoroquinolone antibiotics ciprofloxacin and norfloxacin inhibit bacterial DNA gyrase (a type II topoisomerase) or topoisomerase IV, inhibiting DNA replication and transcription. The aminoglycoside antibiotic kanamycin interacts with the 16S rRNA component of the prokaryotic 30S ribosome subunit, contributing to tRNA mismatching and protein mistranslation and therefore causing the inhibition of protein synthesis [23]. Vancomycin inhibits cell‐wall biosynthesis in gram‐positive bacteria by specifically binding to the d‐Ala‐d‐Ala terminal of the cell‐wall precursor pentapeptide, thus inhibiting transpeptidase‐catalyzed cross‐linking and maturation of the bacterial cell wall (Yarlagadda *et al*., 2016). Thus, vancomycin is the drug of last resort for gram‐positive bacterial infection and is the most commonly used antibiotic to treat MRSA infections. However, due to the increasing use of vancomycin, clinical MRSA isolates with reduced susceptibility to vancomycin have emerged recently [24, 25]. A third-generation cephalosporin, cefotaxime exerts its antibacterial activity by inhibiting cell wall synthesis via blocking the cross-linking of peptidoglycans. Bacteria eventually lyse due to the activities of cell wall autolytic enzymes; therefore, cefotaxime is considered to be bactericidal [26, 27]. We screened substances that use a variety of antibiotics to induce synergy. In our evaluation, chloramphenicol and vancomycin, which acts within gram-positive bacteria, exhibit synergistic action when co-treated with periplanetasin-2. As a result, their antibacterial spectrum has been expanded. Following this finding, research was conducted on the mechanism of action that creates this synergy effect.

The mechanisms of antibacterial agents vary and include the disruption of membrane structure and function as well as the inhibition of DNA replication, protein synthesis, and energy metabolism. Recent studies have shown that the initial interactions of antibiotics with their targets cannot fully explain the lethality of antibiotics and that these interactions induce ROS generation, which contributes to bacterial cell death [28]. Similar to eukaryotic apoptosis, bacterial cell death exhibited apoptosis-like features in the presence of periplanetasin-2 by inducing elevation of intracellular ROS levels and lipid peroxidation [8, 9]. These characteristics cause overexpression of *recA* protein, which acts as a caspase, and intracellular damage caused by over-generated ROS. The SOS response is triggered when bacterial cells are exposed to an antimicrobial agent or environment that triggers DNA damage. Under non-oxidative conditions, *recA* protein regulates the SOS reaction and responds to single-strand DNA damage. However, when the DNA is damaged in an oxidative environment, the structure of *recA* changes, facilitating its action as a caspase-like protein. The deletion of the *recA* gene resulted in a reduction in the apoptosis-like processes that occur in response to antibiotic treatment [29]. Antibiotics showing synergistic effects by periplanetasin-2 induced oxidative damage caused by ROS accompanied by the overexpression of *recA* as caspase.

The maintenance of intracellular cation concentrations and the subsequent controlled ionic gradients across cell membranes are essential for cellular energetics and membrane potential. Furthermore, cellular divalent cation concentrations remain constant over time as they are maintained above the appropriate level for the role of a cofactor for various cellular proteins and enzymes. Therefore, perturbations in intracellular divalent cation gradients can lead to cellular dysfunction and membrane depolarization. Divalent cations are important for maintaining bacterial cellular homeostasis and viability to include differentiation, transcription, transport, pathogenicity, and stabilizing macromolecular complexes and membranes [30, 31]. Calcium ion activity changes according to combination with chloramphenicol and vancomycin. Combination with vancomycin elevates the intracellular calcium level, whereas combination with chloramphenicol does not elevate the calcium level. This means chloramphenicol and vancomycin exert different modes of action. Disruption of calcium ion homeostasis leads to lethal cellular processes, eventually resulting in cell death. Calcium-dependent membrane depolarization is one of the known phenomena induced by variation in calcium concentration [32]. Moreover, in many cases, elevated calcium levels affect cells in an ROS-independent manner, contributing to activation of caspases right away [33]. Excessive influx of calcium ion is therefore able to trigger apoptosis under the influence of antimicrobial agents. Combination with vancomycin may induce apoptosis-like death by stimulating calcium signaling. On the other hand, combination with chloramphenicol independently induces apoptosis-like death by calcium concentration. Cell filamentation, generally a marker of stress, was also observed [34]. When DNA is damaged, the repair process mechanism goes into action. Cell division arrests during operation, causing cell alteration resulting in filamentous phenotypes [35]. Treatment with periplanetasin-2 and vancomycin delayed cell division due to DNA damage and led to filamentation. However, in combination with chloramphenicol, cell division was not observed, suggesting that the mechanism of action of the synergistic effects of the two antibiotics exhibits different patterns.

In conclusion, periplanetasin-2 showed synergistic action when combined with vancomycin and chloramphenicol, with a decrease in the MIC values of the drugs. Periplanetasin-2 has potent antibacterial activity and its mechanism of action in bacterial cells involves a number of targets, including the accumulation of ROS, induction of apoptosis-like death, and impaired cell membrane permeability. These results indicate that periplanetasin-2 is a promising option to improve therapeutic efficacy when used with other common drugs that inhibit protein synthesis or cell-wall synthesis. Due to the distinct characteristic action of periplanetasin-2, novel antimicrobial peptides can be designed that are difficult for yeasts to challenge. Periplanetasin-2 can be used with a narrow spectrum of antibacterial drugs to eliminate pathogenic bacteria, but further exploration is required to determine the treatment potential.

## Figures and Tables

**Fig. 3:**
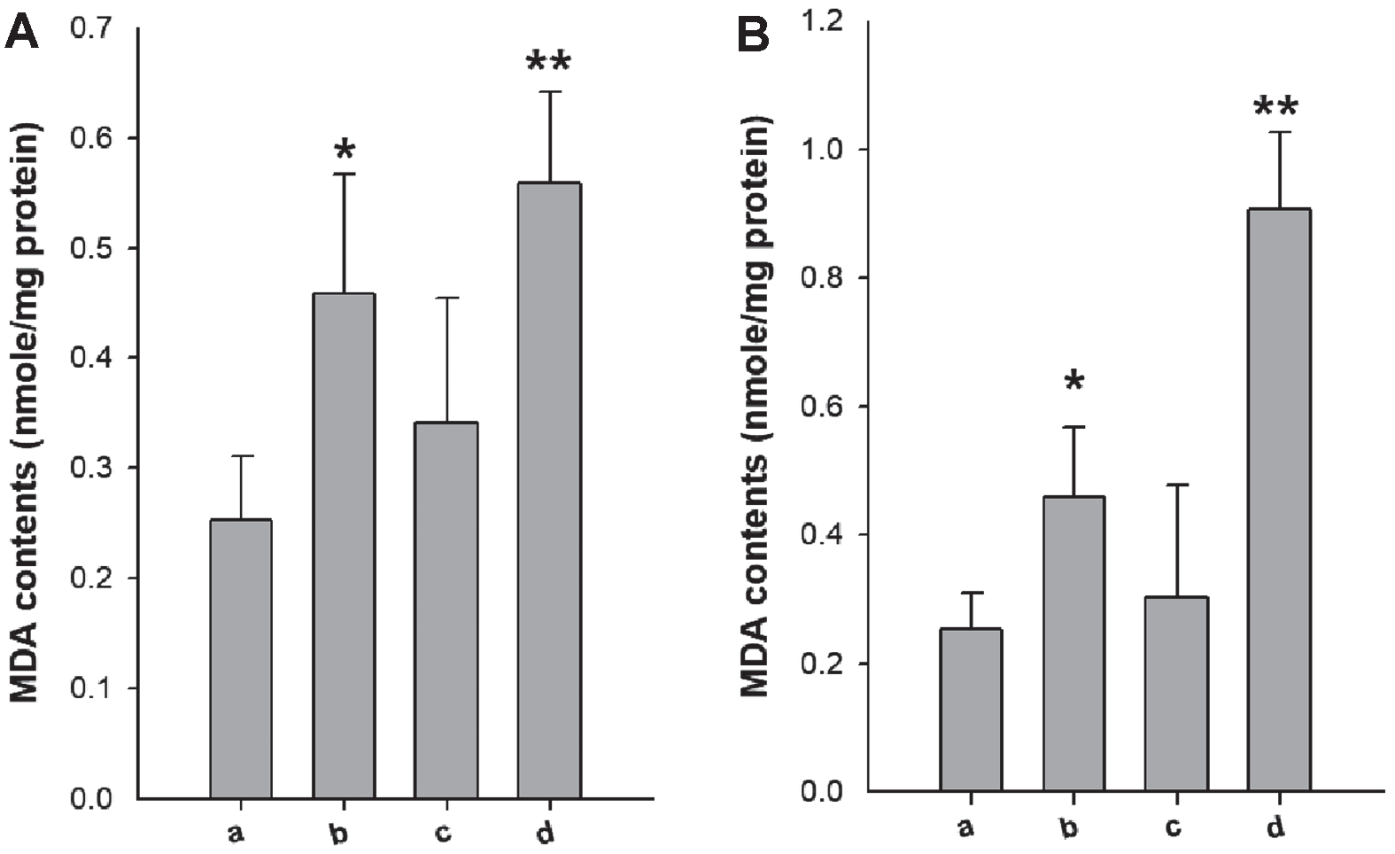
Spectrophotometer analysis of lipid peroxidation in *E. coli*. Lipid peroxidation was determined based on MDA levels. (**A**) (a) Untreated (b) Periplanetasin-2 (c) Vancomycin (d) Periplanetasin-2+vancomycin (**B**) (a) Untreated (b) Periplanetasin-2 (c) Chloramphenicol (d) Periplanetasin-2+chloramphenicol.

**Table 1:** The antibacterial activity of periplanetasin-2 and conventional antibiotics.

*Escherichia coli* BW25113	MIC (μg/ml)
Periplanetasin-2	8
Norfloxacin	1-2
Chloramphenicol	64
Vancomycin	128-256
Amikacin	4
Kanamycin	16
Rifampicin	4-8
Ciprofloxacin	1
Cefotaxime	4

**Table 2:** The combinational activity of periplanetasin-2 and antibacterial agents.

Antibacterial agents	Periplanetasin-2 MIC for combination (μg/ml)	Individual MIC for combination (μg/ml)	FICI^a^	Effect
Vancomycin	1	8	0.156	Synergy
Chloramphenicol	1	16	0.375	Synergy
Norfloxacin	4	1	0.503	No interaction
Ciprofloxacin	1	2	2.125	No interaction
Amikacin	1	16	4.25	Antagonism
Kanamycin	8	16	2	No interaction
Rifampicin	1	8	1.125	No interaction
Cefotaxime	1	2	0.625	No interaction

^a^The fractional inhibitory concentration index (FICI) was calculated by the formula: FICI = (MIC_Drug A_ in combination/MIC_Drug A_ alone) + (MIC_Drug B_ in combination/MIC_Drug B_ alone).
